# Gastric Electrical Stimulation Decreases Gastric Distension-Induced Central Nociception Response through Direct Action on Primary Afferents

**DOI:** 10.1371/journal.pone.0047849

**Published:** 2012-12-20

**Authors:** Wassila Ouelaa, Ibtissem Ghouzali, Ludovic Langlois, Serguei Fetissov, Pierre Déchelotte, Philippe Ducrotté, Anne Marie Leroi, Guillaume Gourcerol

**Affiliations:** 1 Nutrition, Gut & Brain Unit (ADEN – INSERM U1073), Institute for Biomedical Research and innovation, Rouen University, Rouen, France; 2 Department of Nutrition, Rouen University Hospital, Rouen, France; 3 Department of Gastroenterology, Rouen University Hospital, Rouen, France; 4 Department of Physiology, Rouen University Hospital, Rouen, Rouen, France; University of Cincinnati, United States of America

## Abstract

**Background & Aims:**

Gastric electrical stimulation (GES) is an effective therapy to treat patients with chronic dyspepsia refractory to medical management. However, its mechanisms of action remain poorly understood.

**Methods:**

Gastric pain was induced by performing gastric distension (GD) in anesthetized rats. Pain response was monitored by measuring the pseudo-affective reflex (e.g., blood pressure variation), while neuronal activation was determined using c-fos immunochemistry in the central nervous system. Involvement of primary afferents was assessed by measuring phosphorylation of ERK1/2 in dorsal root ganglia.

**Results:**

GES decreased blood pressure variation induced by GD, and prevented GD-induced neuronal activation in the dorsal horn of the spinal cord (T9–T10), the nucleus of the solitary tract and in CRF neurons of the hypothalamic paraventricular nucleus. This effect remained unaltered within the spinal cord when sectioning the medulla at the T5 level. Furthermore, GES prevented GD-induced phosphorylation of ERK1/2 in dorsal root ganglia.

**Conclusions:**

GES decreases GD-induced pain and/or discomfort likely through a direct modulation of gastric spinal afferents reducing central processing of visceral nociception.

## Introduction

Over the past decade, gastric electrical stimulation (GES) has become a new therapeutic option for patients with medically refractory dyspeptic symptoms, including nausea and vomiting, epigastric pain, gastric fullness and early satiety [1]–[9]. This technique is performed through two electrodes inserted to the antrum and connected to a stimulator implanted in the abdominal wall, to deliver bipolar high frequency (14 pulse.min^−1^) low energy (330 µs pulse width) electrical stimulation [10]. GES was initially tested to relieve gastroparesis-related symptoms though the acceleration of gastric emptying, but convergent evidence in humans [3], [4], [11], [12] and in animal models [13] found that gastric emptying remained unchanged after GES, contrasting with the symptomatic improvement. To date, efficacy of GES has been established in one controlled blinded study [2] and is therefore FDA-approved, although GES mechanisms of action are not fully understood.

Recently, our group identified using rodent models that GES could modulate specific brain nuclei involved in the visceral sensory processing, using a post-operative ileus rat model [14]. We showed that GES could act on neurons of the hypothalamic paraventricular nucleus (PVN) to decrease corticotropin-releasing factor (CRF) transcript expression [14]. Interestingly, this effect remained unaltered after subdiaphragmatic vagotomy, suggesting that spinal afferent pathway was likely to be involved. This prompted us to the present study investigating pathways through which GES acts on the brain. We therefore assessed the effect of GES on gastric visceral sensitivity, and determined whether this effect recruited spinal primary afferents. This was achieved by investigating the influence of GES on functional and molecular markers of pain response to gastric distension (GD) in a rat model.

## Materials and Methods

### Animals

Male Sprague-Dawley rats (350–450 g; Janvier, Le Genest-St-Isle, France) were housed in an animal facility that was maintained at 22°C with an automatic 12-hour light/dark cycle. The rats had free access to standard rat chow (RM1 diet; SDS, Witham, Essex, UK) and drinking water. Animals were deprived of food but not tap water 18 h before each experiment. All experiments were performed in anaesthetized rats using sodium thiobutabarbital (Inactin ®, Sigma, Steinheim, Germany) at a dose of 200 mg/kg, given intraperitoneally (ip).

### Ethics Statement

The protocol was approved by the Committee on the Ethics of Animal Experiments of the University of Rouen (Ethical agreement Number: 1008-01). All surgery was performed under sodium thiobutabarbital anesthesia, and all efforts were made to minimize suffering.

### Gastric distension in rats

A spherical infinitely compliant distension balloon (diameter: 3 cm; maximum volume 12 mL) was made using a polyethylene bag attached to a tube in polyethylene 50 mm in diameter (Dutscher, Brumath, France) drilled in its extremity. The balloon was inserted in fasted anaesthetized rats through an incision at tip of the proximal stomach. The balloon was then connected to an electronic barostat (G&J Electronics Inc, Toronto, Canada) to perform isobaric graded GD.

### Visceral pain measurement in anesthetized rats

**Figure 1:**
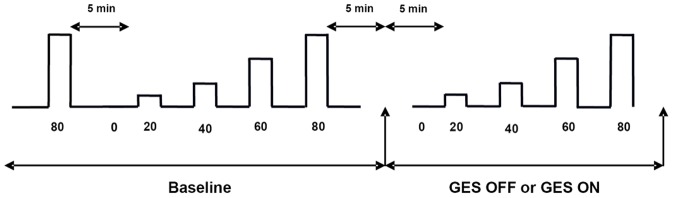
Plan of the experimental protocol of the gastric distensions at 20, 40, 60, 80 mmHg.

The visceral pain was assessed by monitoring the pseudoaffective reflex i.e., cardiovascular changes induced by nociceptive stimuli, and was quantified using the variation of the arterial blood pressure (BP) in response to GD [15]. The BP was measured continuously in anesthetized rats using a perfused catheter (NaCl 0.9%; heparin 0.3%) introduced into the right carotid and connected to a pressure transducer (Solal, Strasbourg, France). Variation of BP was quantified after graded GD at 20, 40, 60, 80 mm Hg (Fig. 1).

### Acute GES in anesthetized rats

Two electrodes (Model no. 4300; Medtronic, Boulogne, France) were implanted into the great curvature of the gastric antrum, approximately 1 cm above the pylorus and then were connected to an external stimulator (Enterra; Medtronic, Boulogne, France). GES was performed using parameters used for the treatment of dyspepsia and gastroparesis (frequency 14 Hz; intensity 5 mA; pulse duration 330 µs; cycle ON 0.1 s; cycle OFF 5.0 s). The sham stimulation group (OFF) underwent the same surgical procedure but the electrodes were not connected to the stimulator.

### C-fos immunohistochemistry

C-fos immunochemistry was performed after 2 h of GD at 60 mmHg (20 sec of distension every 4 min for 2 h) and/or stimulation, according a previous method [16]. Briefly, anesthetized rats were perfused through a cardiac-aorta cannula with saline followed by 200 ml/rat of ice-cold 4% paraformaldehyde and 14% saturated picric acid in 0.1 M phosphate buffer solution (pH 7.2). After decapitation, the brain and thoracic spinal cord (T9–T10) were post-fixed in the same fixative solution 2 h and cryoprotected by immersion in 10% sucrose overnight, and transferred to 30% sucrose for one day. The brain, brainstem and T9–T10 segment were transversally sectioned at 25 µm with a cryostat. Sections were incubated with rabbit anti-c-fos (Calbiochem, Darmstadt, Germany) at 1∶2000 dilution (PBS, Triton 0.3%, NaAzide 0.01%) overnight at 4°C, and after several washes, were incubated in Cy3-conjugated goat anti-rabbit IgG at 1∶400 (Fisher, Invitrogen, Carlsbad, CA) for two hours at room temperature. The number of c-fos-immunoreactive (IR) nuclei in the PVN (1.7 mm caudal to Bregma), the NTS (13.7 mm caudal to Bregma) and in lamina I, II and III of dorsal spinal cord was counted bilaterally in 3 sections randomly selected per rat and expressed as the mean number of c-fos-IR within left and right nuclei (n = 4–10 per group). For double staining of c-fos stained sections in the brain, we used guinea pig polyclonal antibody against CRF (1∶400; Santa Cruz Biotechnology, Santa Cruz, CA).

### Phosphorylated ERK½ immunochemistry in DRGs

Phosphorylated ERK½ (pERK½) immunochemistry was performed after 5 min of GD and/or stimulation. DRGs (T9–T10) were dissected out and fixed as detailed above, then sectioned at 8 µm with cryostat, and post-fixed for 20 min with fixative solution. Sections were permeabilized with 0.2% triton in phosphate-buffered saline (PBS; 0.01 M, pH 7.4, 0.25% Tween 20) for 5 min, blocked with 5% albumin bovine serum (Sigma-Aldrich, St-Louis, MO) in PBS, and incubated overnight with primary antibody in PBS: mouse anti-pERK (1∶100, Cell Signaling, Saint Quentain-en-Yvelines, France). After several rinses with PBS, sections were incubated with the secondary antibody (Cy3-conjugated donkey anti-mouse antibody; 1∶250 in PBS; Jackson, West grove, PA) for 2 hrs, rinsed, mounted, and coverslipped. Three sections were analyzed per rat (n = 6 per group) and the number of pERK½ immunoreactive cells was counted and expressed as the percentage of positive cells to the total cells in each ganglion.

### Experimental protocols

#### Effect of GES on variation of BP to graded GDs in anesthetised rats

In fasted anesthetised rats, a first set of graded GD was performed, followed 15 min later by a second series of GD under GES (ON group, n = 10) or sham GES (OFF group, n = 9). In the ON group, GES was started 5 min prior the second set of GD. In one group, electrical stimulation was performed by implanting the electrode into the caecum instead of the stomach wall and using the same stimulation parameters (caecal stimulation group; n = 7). To assess the involvement of opioidergic system in the effect of GES, naloxone (1 mg/kg, Mylan, Chambray-Les-Tours, France) or saline was injected iv 2 min before the start of GES (n = 8–9/group).

#### Influence of GES on GD induced c-fos expression in the CNS of anesthetised rats

C-fos immunochemistry was performed 2 h after GD composed of successive phasic distensions at 60 mmHg (20 s) separated by 240 s, or in the absence of GD (balloon inserted but not inflated). The influence of GES on c-fos immunoreactivity was assessed by performing GES in rats undergoing GD or not, and or sham GES in rats undergoing GD or not (n = 4–10 for all experiments). To assess whether the effect of GES recruited descending input from the supraspinal centres toward the dorsal root ganglia of the spinal cord, the same experimental procedure was conducted in rats with prior spinal cord transection performed at the thoracic metameric T5–T6 level through laminectomy 15 minutes before GES and/or GD.

#### Influence of GES on GD induced ERK½ Phosphorylation in rat DRGs

ERK½ Phosphorylation was assessed in DRGs 5 min after GD composed of successive phasic distensions at 60 mmHg (20 s) separated by 40 s, or in the absence of GD (balloon inserted but not inflated). The influence of GES on ERK½ Phosphorylation was assessed by performing GES in rats undergoing GD (n = 6) or not, or sham GES in rats undergoing GD or not.

### Statistical Analysis

Data are expressed as median ± SEM per group or per section. Wilcoxon matched paired test was used to compare the variation of BP between the first and second set of distension. Differences among groups were assessed using one-way ANOVA followed by Bonferronni's multiple comparison tests or using Mann and Whitney test for inter-individual pair wise comparisons. A p-value of <0.05 was considered statistically significant.

## Results

### Gastric electrical stimulation decreases the variation of blood pressure in response to gastric distension

**Figure 2:**
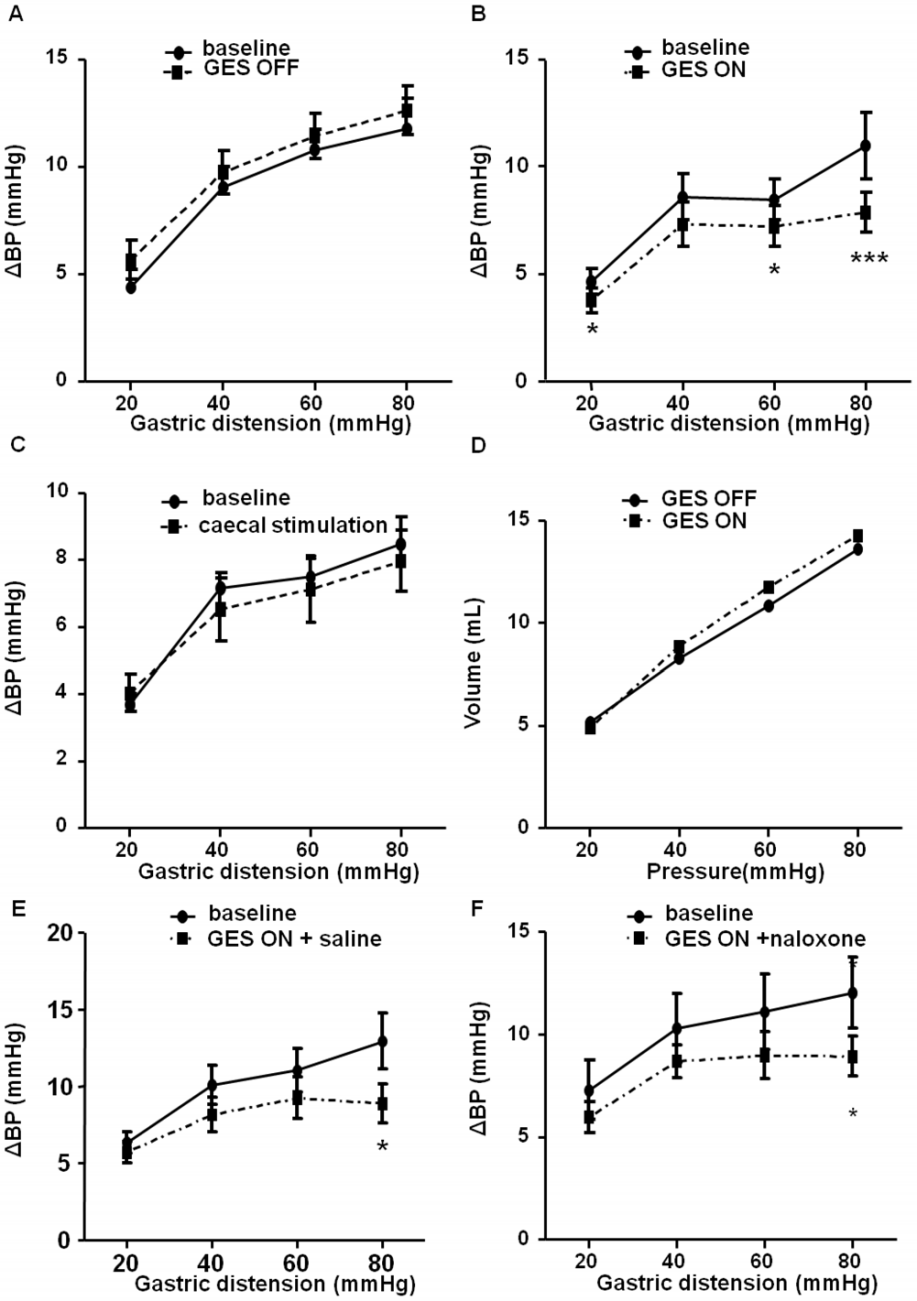
Effect of gastric electrical stimulation (GES) on the variation of blood pressure (BP). A, B, C. Variation of BP in response to graded gastric distension (GD) of 20, 40, 60, 80 mmHg: a first set of GD was performed without stimulation (Baseline) then followed by a second set of GD, during either sham (OFF; A) or effective electrical stimulation (ON) of the stomach (B) or the caecum (C). D. Variation of the intra-gastric volume according to the intra-gastric pressure during the different levels of gastric distension without (OFF) and under gastric electrical stimulation (ON). E, F. Influence of intravenous pretreatment with saline (D) or naloxone (1 mg/kg; E) on variation of BP during GES. Data are expressed as mean ± SEM of 7–10 animals per group. *: p<0.05, ***: p<0.001.

In an attempt to validate a rat model to assess the effect of GES on gastric visceroception, pseudo-affective reflex, namely the variation of BP in response to GD, was measured as a marker of gastric nociception. In anesthetized rats, basal BP was not significantly modified throughout the experiment, as demonstrated by the absence of variation of basal BP before each GD. By contrast, GD induced a rise in BP during each distension level. Graded GD increased the variation of BP from 4.62±0.61 mmHg at 20 mmHg of distension to 10.95±1.54 mmHg at 80 mmHg of distension. The variation of BP remained unaffected after sham GES in the OFF group (Fig. 2A). By contrast, gastric but not caecal electrical stimulation decreased the variation of BP compared to the first set of distension (Fig. 2B–C). This effect was not related to modification of gastric compliance that remained comparable in the OFF and ON groups (Fig. 2D). In addition, the GES-induced decrease of BP variation in response to GD was not prevented by iv injection of naloxone (Fig. 2E–F).

### Gastric electrical stimulation prevents gastric distension-induced rise in c-fos in the dorsal horn of the spinal cord, in the NTS and the PVN

**Figure 3:**
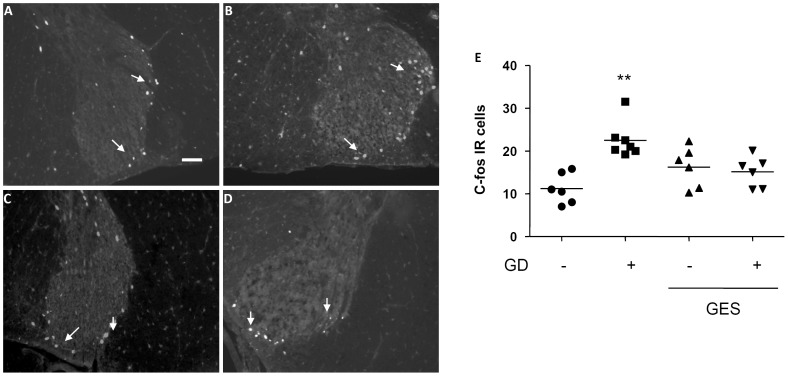
Effect of gastric electrical stimulation (GES) on c-fos expression in the T9/T10 dorsal horn of spinal cord. A, B, C, D. Representative photomicrographs illustrating expression of c-fos protein in the dorsal horn of spinal cord following either gastric distension (GD; 60 mmHg for 2 hours; B, D) or sham GD (A, C) during GES (C, D) or sham GES (A, B). Scale bar = 100 µm. E. Quantification of the number of c-fos immunoreactive cells in the dorsal horn of spinal cord with the median shown as a black line. **: p<0.01 vs the other groups.

A few c-fos positive cells were observed in the dorsal horn of the spinal cord at the T9 level in the animals not distended (11.2±1.5 cells/section; Fig. 3A). By contrast, a robust expression of c-fos protein was observed bilaterally in the T9 thoracospinal cord 2 h after GD (Fig. 3B), mostly in the superficial dorsal horn (laminae I and II), along the lateral collateral visceral afferent pathway (+101%; p<0.05 vs not distended group). GES did not modify the number of c-fos immunoreactive cells in non-distended animals, whereas GES prevented the rise in c-fos reactive cells after GD (Fig. 3D, 3E) at this level (+35%; p<0.05 vs distended group).

**Figure 4:**
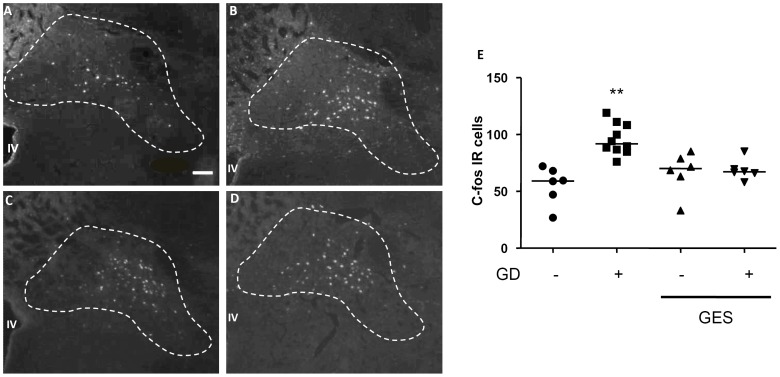
Effect of gastric electrical stimulation (GES) on c-fos expression in the nucleus of the solitary tract (NTS). A, B, C, D. Representative photomicrographs illustrating expression of c-fos protein in the NTS following either gastric distension (GD; 60 mmHg for 2 hours; B, D) or sham GD (A, C) during GES (C, D) or sham GES (A, B). Scale bar = 100 µm. E. Quantification of the number of c-fos immunoreactive cells in the NTS with the median shown as a black line. **: p<0.01 vs the other groups.

**Figure 5:**
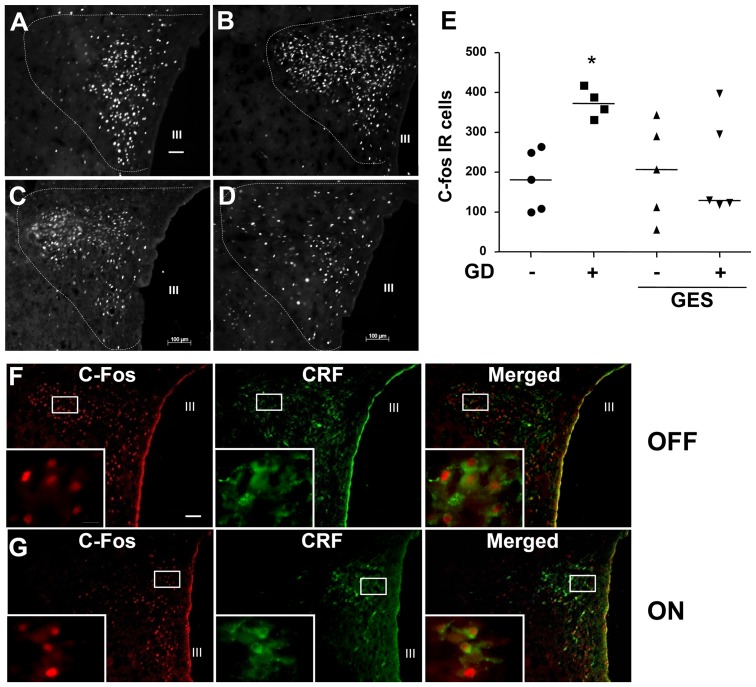
Effect of gastric electrical stimulation (GES) on c-fos expression and CRF expression in the hypothalamic paraventricular nucleus (PVN). A, B, C, D. Representative photomicrographs illustrating expression of c-fos protein in the PVN following either gastric distension (GD; 60 mmHg for 2 hours; B, D) or sham GD (A, C) during GES (C, D) or sham GES (A, B). E. Quantification of the number of c-fos immunoreactive cells in the PVN with the median shown as a black line. F,G. Immunofluorescence double staining of c-fos and CRF in hypothalamic paraventricular nucleus (PVN) during sham (F) or effective gastric electrical stimulation (G) after gastric distension (60 mmHg for 2 h). Scale bars = 100 µm and 10 µm. III: third ventricle.*: p<0.05 vs the other groups.

A similar impact of GD and GES was noted on c-fos expression in the NTS (Fig. 4) and the PVN (Fig. 5). Indeed, GD induced a rise in c-fos immunoreactive cells within the NTS (+72%; p<0.05) and the PVN (+108%; p<0.05) compared to the animals not distended. Likewise, GES had no impact on c-fos expression in non-distended animals (NTS: +20%; p>0.05 vs distended group; PVN: +12%; p>0.05 vs distended group). Conversely, GES prevented the rise in c-fos expression induced by GD in the NTS (+24%; p<0.05 vs distended group) and the PVN (+18%; p<0.05 vs distended group). Further analysis showed that GD induced a marked c-fos expression in CRF-expressing neurons of the PVN (71%) which was prevented by GES (Fig. 5). Scattered c-Fos-IR nuclei were also observed in the area postrema, the arcuate nucleus, the dorsomedial vagus nucleus and locus coeruleus after GD and/or stimulation but without significant differences among groups (data not showed).

### GES effect on the spinal cord does not involve descending projections from supra-spinal centers

**Figure 6:**
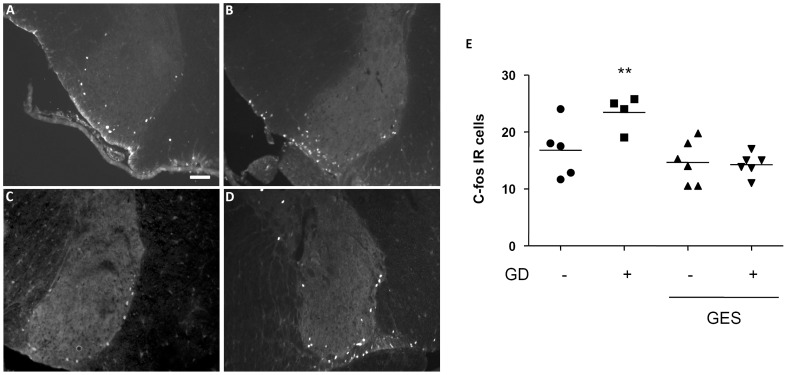
Effect of gastric electrical stimulation (GES) on c-fos expression in the T9/T10 dorsal horn of spinal cord after T5/T6 transection. A, B, C, D. Representative photomicrographs illustrating expression of c-fos protein in the dorsal horn of spinal cord following either gastric distension (GD; 60 mmHg for 2 hours; B, D) or sham GD (A, C) during GES (C, D) or sham GES (A, B). Scale bar = 100 µm. E. Quantification of the number of c-fos immunoreactive cells in the dorsal horn of spinal cord with the median shown as a black line. **: p<0.01 vs the other groups.

**Figure 7:**
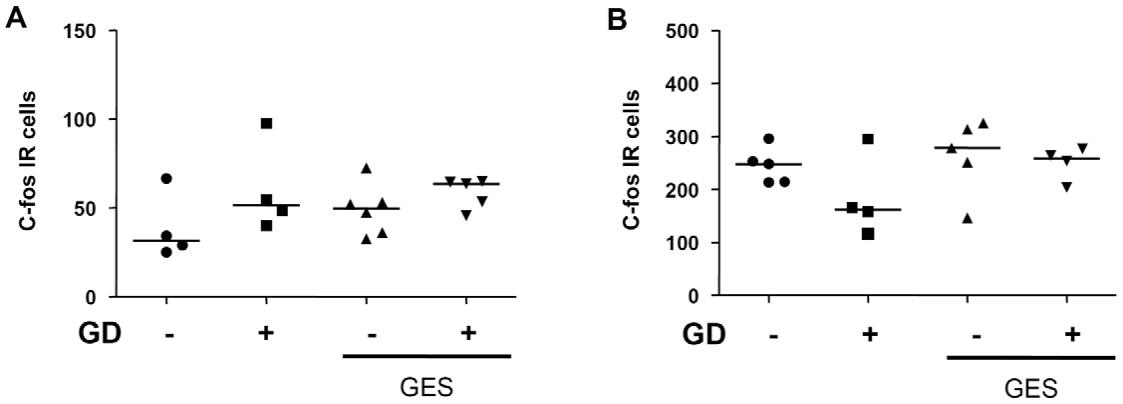
Effect of GES on c-fos expression in the NTS and in the hypothalamic paraventricular nucleus (PVN) after T5/T6 transection. Quantification of the number of c-fos immunoreactives cells in the NTS (A) and in the PVN (B) following either GD (60 mmHg for 2 hours) or sham GD during GES or sham GES. Data are expressed as median ± SEM of 4–6 animals per group.

To determine whether the effect of the GES on c-fos expression within the T9 dorsal horn of the spinal cord involved descending projections from supra-spinal centers, c-fos protein expression was determined after T5–T6 spinal cord transaction (Fig. 6). Compared to the non-distended group, GD provoked an increase in c-fos immunoreactive cells in the dorsal horn of the T9 spinal cord (+40%; p<0.05; Fig. 6A, 6B), with similar distribution as in previous experiments. Interestingly, GES still prevented the rise in c-fos expressing cells at this level after T5–T6 transection (GES non-distended: −15%; GES distended: −13%; p>0.05; Fig. 6C, 6D). By contrast, the effect of GES on c-fos expression within the NTS and the PVN was lost after T5–T6 transection (Fig. 7).

### Gastric electrical stimulation prevents gastric distension-induced phosphorylation of ERK½ in rat dorsal root ganglia

**Figure 8:**
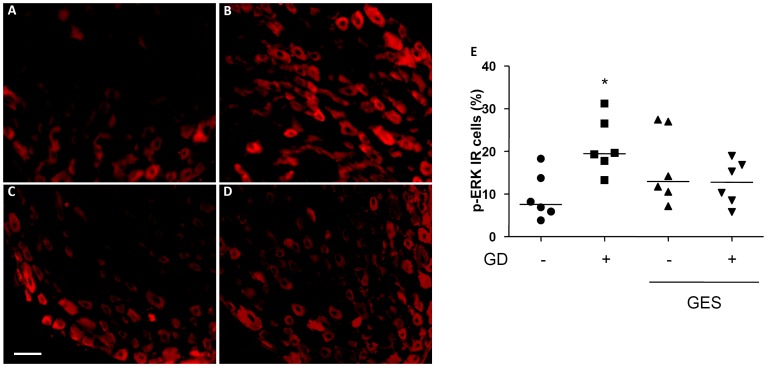
Effect of gastric electrical stimulation (GES) on pERK1/2 expression in T9 dorsal root ganglia. A, B, C, D. Representative photomicrographs illustrating expression of p-ERK1/2 protein in dorsal root ganglia, following either gastric distension (GD; 60 mmHg for 5 minutes; B, D) or sham GD (A, C) during GES (C, D) or sham GES (A, B). Scale bar = 50 µm. E. Quantification of the percentage of p-ERK1/2 immunoreactive cells within each ganglia, with the median shown as a black line. *: p<0.05 vs the other groups.

To assess whether GES could impact or not on gastric distension activated primary visceral afferents, phosphorylation of ERK½ was determined in rat dorsal root ganglia (Fig. 8). Compared to non-distended animals, GD induced a rise in cells immunoreactive for pERK½ (+124%; p<0.05; Fig. 8A, 8B). GES applied 30 min before the start of GD prevented the increase in pERK½ positive cells induced by GD (GES non-distended: +71%; GES distended: +32%; p>0.05; Fig. 8C, 8D).

## Discussion

Although GES has become over the past years a new therapeutic approach in gastroparesis and/or dyspepsia–related chronic vomiting and nausea, its mechanisms of action is not yet fully understood. In the present study, we provide evidence, using a rodent model, that GES impacts on gastric visceroception. In fact, we showed that GES could increase nociceptive thresholds to GD, and modulate brain centers probably via the spinal cord. The prevention of ERK½ phosphorylation in response to GD by GES in dorsal root ganglia suggests that gastric extrinsic primary afferents might be involved in this effect. Although further studies are warranted to establish whether this mechanism is involved in the symptomatic improvement observed after GES or not, our data clearly indicate that GES exerts a sensitive effect on the stomach which is at least correlated with the clinical outcome of implanted patients.

Although early concepts about GES relied on its ability to accelerate gastric emptying, most of the recent studies conducted in patients and animal models found no or little effect of GES using high frequency (e.g.>0.3 Hz) on gastric emptying. This was first demonstrated by Chen et al. who showed, in a canine model of gastroparesis, that GES applied with higher frequency that the natural pacemaker did not modify gastric myoelectrical activity [13]. On the other hand, GES performed with larger impulse width (>0.3 s) and given at a frequency close to the natural gastric pace-maker rhythm (e.g. at 0.05 Hz in Human) could entrain gastric slow waves activity [13]. This was confirmed in cohort studies that showed an absence of gastric emptying acceleration after GES [3], [4], [11], [12]. In addition, symptomatic improvement after GES was not correlated to gastric emptying acceleration [4]. Finally, we and others showed that GES was an effective therapeutic alternative in patients with medically refractory nausea and vomiting despite being in the normal range of gastric emptying before implantation [3], [4], [12]. Altogether, these studies converge to the fact that gastric emptying remains unaffected by GES, and that functional improvement is related to other mechanisms.

To date, mechanisms trough which GES display a sensitive effect remain largely unknown. In fact, one study reported in 10 patients that discomfort pressure and volume thresholds were increased 6 weeks after GES [17].

Therefore, we developed a rodent model of GD-induced visceral nociception using a previously validated method in anesthetized rats [7]. We showed that electrical stimulation of the stomach, but not the caecum, could modulate pseudo-affective response, used as a nociceptive marker. Interestingly, gastric compliance was not affected by GES in our study, indicating that GES acts through a sensitive effect rather than an effect on gastric relaxation. The effect of GES on gastric pain thresholds was further confirmed by measuring c-fos expression within the T9-T10 dorsal horn of the spinal cord that showed that GES prevented the GD-increased c-fos positive cells at this level. Interestingly, the effect of GES on the dorsal horn of the spinal cord remained unaltered after T5–T6 spinal cord transection, suggesting that descending supraspinal pain controls, including diffuse noxious inhibitory controls, were not involved in GES-antinociceptive effect in our model. In addition, naloxone, a non-specific opioïd receptors antagonist, did not alter GES-effect on pseudo-affective reflex suggesting that neither peripheral nor central opioïd pathway were recruited during acute GES. In fact, we showed in our model that GES could prevent GD-induced phosphorylation of ERK1/2 in DRGs. Although semi-quantitative, pERK protein expression using immunochemistry has been used in the past to measure activation of extrinsic primary afferent neurons originating from the gut in response to gut stimuli, and is therefore accurate to detect neuronal activation in response to GD [18], [19]. Therefore, our data suggests that GES acts directly on primary spinal afferent fibers mediating pain.

From these results, two further hypotheses could be drawn. First, we have reported that GES induced ghrelin release from the stomach in rodents, and ghrelin has been shown to act on DRGs glial cells [20] and spinal cord [21] to decrease somatic pain. It is therefore conceivable that GES-induced release of ghrelin increases GD nociceptive thresholds through an endocrine pathway with DRGs as the target site. On the other hand, electrical stimulation, given 5 s prior to noxious stimuli, has been shown to act as a conditioning stimulus to decrease pain transduction from mechano–heat sensitive C-fiber afferents [22]. This phenomena, also called “*fatigue in nociceptors*”, is prominent with electrical, but not heat or mechanical pre-conditioning stimuli [22]. Electrical pulses are usually not perceived by patients undergoing GES, probably because the two electrodes stimulate a few local nociceptive fiber units, when the recruitment of a higher number of fibers with higher intensities is required to elicit spinal and/or central pain transduction. However, GES, by stimulating gastric nociceptive afferents below perceived pain threshold, may prevent larger recruitment of these fibers to more intense nociceptive stimuli such as GD. Altogether, our data indicate for the first time that GES acts on gastric sensitivity through a direct effect on gastric primary afferent, and this effect is likely to be a prerequisite for central action of GES.

In the present study, we also showed that GES could modulate supraspinal centers involved in visceral pain processing. In fact, we previously showed, using a rodent model of post-operative ileus, that GES could prevent the increase of c-fos expression induced by the ileus within the NTS and the PVN in catecholaminergic and CRF neurons, respectively [23], [14]. This was corroborated by Tang et al., who showed that GES in rat inhibited extracellular potentials of PVN neurons [24]. In agreement with these previous studies, we showed in the present work that GES could also prevent the rise in c-fos expression in the NTS and the PVN in response to another visceral stimulus, namely the phasic isobaric distension of the stomach. To our knowledge, this is the first report showing that CRF neurons of the PVN are activated during isobaric GD. In agreement with previous studies, we also showed that GES decreases c-fos expression in CRF neurons of the PVN in response to GD. In fact, CRF neurons in the PVN play a pivotal role in the induction of emesis and the decrease in food intake during various visceral inflammatory stimuli, including post-operative ileus and endotoxemic shock [25], [26]. Furthermore, central CRF is known to play a pivotal role in chronic stress-related pain induction and maintenance through CRF_1_ receptor pathway [27]. Therefore, long term effect of GES on gastric nociception modulation might involve central CRF by decreasing central CRF release and thus CRF_1_ pathway activation. Whether PVN CRF neurons are modulated during chronic GES in patients and are involved in symptomatic improvement after GES therapy remains to be investigated. Using brain functional imaging, McCallum R et al., showed that GES could modulate thalamic and caudate nuclei in implanted patient, suggesting thus that GES may also exert a sensitive effect in humans [17].

## Conclusions

In conclusion, we showed that GES could alleviate gastric pain and/or discomfort induced by GD, both in patient and in rats by decreasing central processing of visceral nociception. This effect is likely to be related to direct action on gastric spinal afferents, the nature of which remaining to be determined.
